# Thromboembolic complications were not different between intravenous and epidural analgesia after unilateral knee arthroplasty under neuraxial anesthesia: a propensity-score matched analysis

**DOI:** 10.1186/s13741-026-00643-y

**Published:** 2026-01-17

**Authors:** Ja Eun Lee, Soo Joo Choi, Mi Sook Gwak, Dae Kyun Ryu, Jaekyeong Song, Sook Young Woo, Young-Wan Moon, Ji Won Choi

**Affiliations:** 1 Department of Anesthesiology and Pain Medicine, Samsung Medical Center, Sungkyunkwan University School of Medicine, 81 Irwon-ro, Gangnam-gu, Seoul, 06351 Republic of Korea; 2 Department of Statistics and Data Center, Samsung Medical Center, Seoul, Republic of Korea; 3 Department of Orthopedic Surgery, Samsung Medical Center, Sungkyunkwan University School of Medicine, Seoul, Republic of Korea

**Keywords:** Arthroplasty, Replacement, knee, Thromboembolism, Anesthesia, regional, Analgesia, epidural, Analgesia, Patient-Controlled

## Abstract

**Background:**

We aimed to compare thromboembolic (TE) complications between intravenous and epidural analgesia after unilateral total knee arthroplasty (TKA) under neuraxial anesthesia.

**Methods:**

In this retrospective study, patients who received spinal anesthesia (SA) and intravenous patient-controlled analgesia (IV-PCA) were allocated to the SA-IV group, and those who received combined spinal-epidural (CSE) anesthesia and epidural PCA were allocated to the CSE-E group. Primary outcome was composite incidence of in-hospital TE events defined as myocardial infarction, stroke, peripheral artery occlusion, pulmonary embolism, or deep vein thrombosis. Secondary outcomes were general complications and pain score. After propensity score matching, outcomes were compared using generalized estimating equation.

**Results:**

Among 1,244 cases from 2016 to 2022 at a tertiary hospital, 321 patients in SA-IV and 214 patients in CSE-E were analyzed after matching. The incidence of TE complications was comparable between SA-IV and CSE-E groups [0.9% (*n* = 3) vs. 2.8% (*n* = 6); odds ratio (OR) 1.88, 95% confidence interval (CI) 0.89–10.57; *p* = 0.08]. There were no differences in general complications, delirium, falls, or bedsores. In the CSE-E group, transient motor weakness was more frequent (OR 2.70, 95% CI 9.27-451.78; *p* < 0.001), and the number of days to joint exercise initiation was higher. However, pain score after TKA was significantly lower in the CSE-E group [5(3–6) vs. 3(2–5); *p* < 0.001].

**Conclusion:**

In this retrospective analysis, the incidence of TE complications after TKA under neuraxial anesthesia was not significantly different between intravenous and epidural analgesia. Epidural analgesia was associated with lower pain intensity, higher incidence of motor weakness, and late initiation of exercise.

## Background

Total knee arthroplasty (TKA) offers relief of chronic pain and improved quality of life to patients with end-stage knee osteoarthritis (Kahlenberg et al. 2018; Canovas and Dagneaux 2018). TKA demand has been exploding worldwide due to extended life expectancy, with continued growth expected for several more decades (Inacio et al. 2017; Kim et al. 2021; Culliford et al. 2015). However, major arthroplasties carry risks of thromboembolic (TE) complications, which can increase medical cost, prolong hospital stay, and result in mortality (Shahi et al. 2018; Baser et al. 2011; Keller et al. 2020). A recently published expert consensus by the International Consensus on Anaesthesia-Related Outcomes after Surgery (ICAROS) group concluded that, in primary knee arthroplasties, neuraxial anesthesia has advantages over general anesthesia with regard to TE complications (Memtsoudis et al. 2019). Neuraxial anesthesia includes spinal, epidural, and combined spinal-epidural blocks, which have become the mainstay of anesthesia for lower extremity joint arthroplasty in place of general anesthesia (Rodriguez-Patarroyo et al. 2021).

Notably, use of an epidural route can extend into the postoperative period and provide continuous analgesia with an indwelling catheter, which has been reported to reduce TE complications compared with systemic analgesia following general anesthesia (Moraca et al. 2003; D’Ambrosio et al. 2015). However, recent evidence suggests that this role of epidural analgesia needs to be reconsidered (Rune and Hasselager 2022; Stavros et al. 2021; Luo et al. 2025). Yet there is very limited data looking at whether postoperative epidural analgesia would further reduce TE complication rate when comparing with systemic analgesia. Furthermore, epidural analgesia can lead to urinary retention, pruritus, hypotension and more serious complications like neurologic injury, epidural hematoma, and infection, requiring labor-intensive monitoring (Rawal 2012, 2021).

Therefore, in this retrospective propensity-score matched (PSM) study, we aimed to compare the incidence of TE events after primary unilateral TKA between spinal anesthesia followed by postoperative intravenous analgesia and combined spinal-epidural anesthesia followed by postoperative epidural analgesia. We hypothesized that the incidence of thromboembolic complications would be different between the intravenous analgesia group and the epidural analgesia group.

## Methods

### Study population and design

This retrospective study on primary unilateral TKA under neuraxial anesthesia performed between January 2016 and December 2022 at our tertiary teaching hospital was conducted under the approval of the Institutional Review Board of Samsung Medical Center, Seoul, Republic of Korea (file number: 2023-10-012) and according to the principles of Declaration of Helsinki. Clinical information was extracted from our electronic medical records (EMR) system. For data that could not be extracted, investigators in this study reviewed the EMR directly.

Patients who received spinal anesthesia (SA) and postoperative intravenous patient-controlled analgesia (IV-PCA) were allocated to the SA-IV group, and those who received combined spinal-epidural anesthesia (CSE) and postoperative epidural PCA (PCEA) were allocated to the CSE-E group. Exclusion criteria were staged bilateral surgery within the same admission, missing data, and conversion to general anesthesia.

### Outcome measures

The primary outcome was the composite incidence of in-hospital arterial and venous TE events defined as myocardial infarction, ischemic stroke, pulmonary embolism, and deep vein thrombosis confirmed by specialists in the relevant departments and with imaging modalities such as coronary angiography, brain magnetic resonance imaging, computed tomography pulmonary angiography, and ultrasonography. Secondary outcomes were general complications [delirium; care-related problems (falls, bedsores); and metabolic (renal/hepatic insufficiency or failure), pulmonary (pneumonia, pulmonary edema, pleural effusion, atelectasis), or cardiac (cardiac arrest, heart failure, new onset arrhythmia, new onset atrial fibrillation) complications], unwanted neurologic symptoms (new onset or aggravated lower extremity paresthesia, motor weakness, numbness, back pain, and other related symptoms), days to initiation of range of motion (ROM) exercise, and pain at the peak numerical rating scale (NRS) score (0, no pain; 10, worst pain imaginable) on the first postoperative day (POD1) and the first day of ROM exercise (ROM1). As previously described, all TE events were diagnosed by comprehensively assessing symptoms and signs of patients, imaging studies, and related medical department consultation history.

### Anesthetic procedures

For SA, patients were positioned in the lateral decubitus position, and lumbar interlaminar spaces were identified by surface landmarks. After skin sterilization and local infiltration, a 25G Whitacre needle was inserted into one of L3/4, L4/5, or L5/S1 spaces and advanced via the midline or paramedian approach until reaching the intrathecal space as confirmed by clear and free-flowing cerebrospinal fluid. A single-shot injection of 0.5% bupivacaine 9 to 13 mg and morphine sulfate 100 to 200 µg was performed. After injection, patients were positioned supine and the level of block was tested, permitting surgical preparation after confirming sensory blockade of at least T12 and motor blockade of Bromage grade 2 (unable to raise extended leg and move knee) or higher.

For CSE, all procedural details were the same as SA until intrathecal injection, after which an additional step of inserting an epidural catheter was performed. An 18G Tuohy needle was inserted at one of the L2/3, L3/4, or L4/5 spaces and advanced with the loss-of-resistance technique. After entering the epidural space, the catheter was inserted and fixed to the skin with occlusive dressing. If catheter insertion failed by the above surface landmark-guided technique or was deemed difficult due to anatomical issues, fluoroscopy-guided insertion was conducted either preoperatively or postoperatively at our outpatient pain management clinic. During surgery, both groups were sedated under continuous infusion of propofol and remifentanil.

### Postoperative analgesia regimen

The IV-PCA regimen was fentanyl 20 µg/ml with a bolus of 0.5 ml, lock-out time of 15 min, and a basal infusion rate of 0.5 ml/hr. The PCEA regimen was ropivacaine 1.5 mg/ml and hydromorphone 4 µg/ml with a bolus of 2 ml, lock-out time of 15 min, and a basal infusion rate of 4 ml/hr. Patients on PCA were monitored for possible side effects (respiratory depression, pruritus, nausea and vomiting, hypotension, dizziness) and managed accordingly. Oral analgesics (non-opioids and/or opioids) were given daily until discharge. In the CSE-E group, most epidural catheters were removed on the third POD.

### Postoperative patient management

After surgery, the patients were monitored regularly for neurologic and circulatory changes of the lower extremity as well as for side effects of IV-PCA or PCEA in the ward. Standard physical therapy was supervised by physiotherapists. All patients received routine mechanical prophylaxis including ankle pump exercises, intermittent pneumatic compression, and anti-embolic stockings.

### Statistical analysis

A two-sample T-test or Wilcoxon’s rank sum test was used for continuous variables according to normality. Normality was tested with the Shapiro-Wilk or Kolmogorov-Smirnov test. For categorical variables, chi-square or Fisher’s exact test was used and odds ratio (OR) and 95% confidence interval (CI) from logistic regression were calculated. Because there were potential confounding factors for the primary outcome, propensity score matching to balance the following variables was performed: age, sex, American Society of Anesthesiologists (ASA) physical status classification (I-II vs. ≥III), body mass index, diabetes mellitus, hypertension, preoperative consumption of an antithrombotic (anticoagulant and/or antiplatelet) for underlying medical disease, preoperative serum C-reactive protein (CRP) concentration, operation site (left or right knee), operation time, and intraoperative estimated blood loss. The propensity score was estimated from a multivariable logistic regression model, and 2:1 nearest neighbor matching without replacement was used with a caliper of 0.05 in which the ratio argument determined the maximum number of controls to match each treated unit. Absolute standardized mean differences less than 15% were considered balanced. After matching, outcomes were compared using generalized estimating equations (GEE), and OR with 95% CI was denoted for categorial outcomes. For sensitivity analysis ensuring double robustness, we performed an additional GEE analysis on the matched cohort by further adjusting for the propensity score as a covariate. All tests were two-tailed, with *p*-value < 0.05 considered statistically significant, as corrected by Bonferroni’s method in cases of multiple tests. Given the low incidence of TE events observed in this study, the statistical power to detect significant differences between groups may be limited. Therefore, the possibility of a Type II error should be considered when interpreting the non-significant findings regarding the primary outcome. Statistical analyses were performed with R 4.1.0 (Vienna, Austria).

## Results

**Fig. 1:**
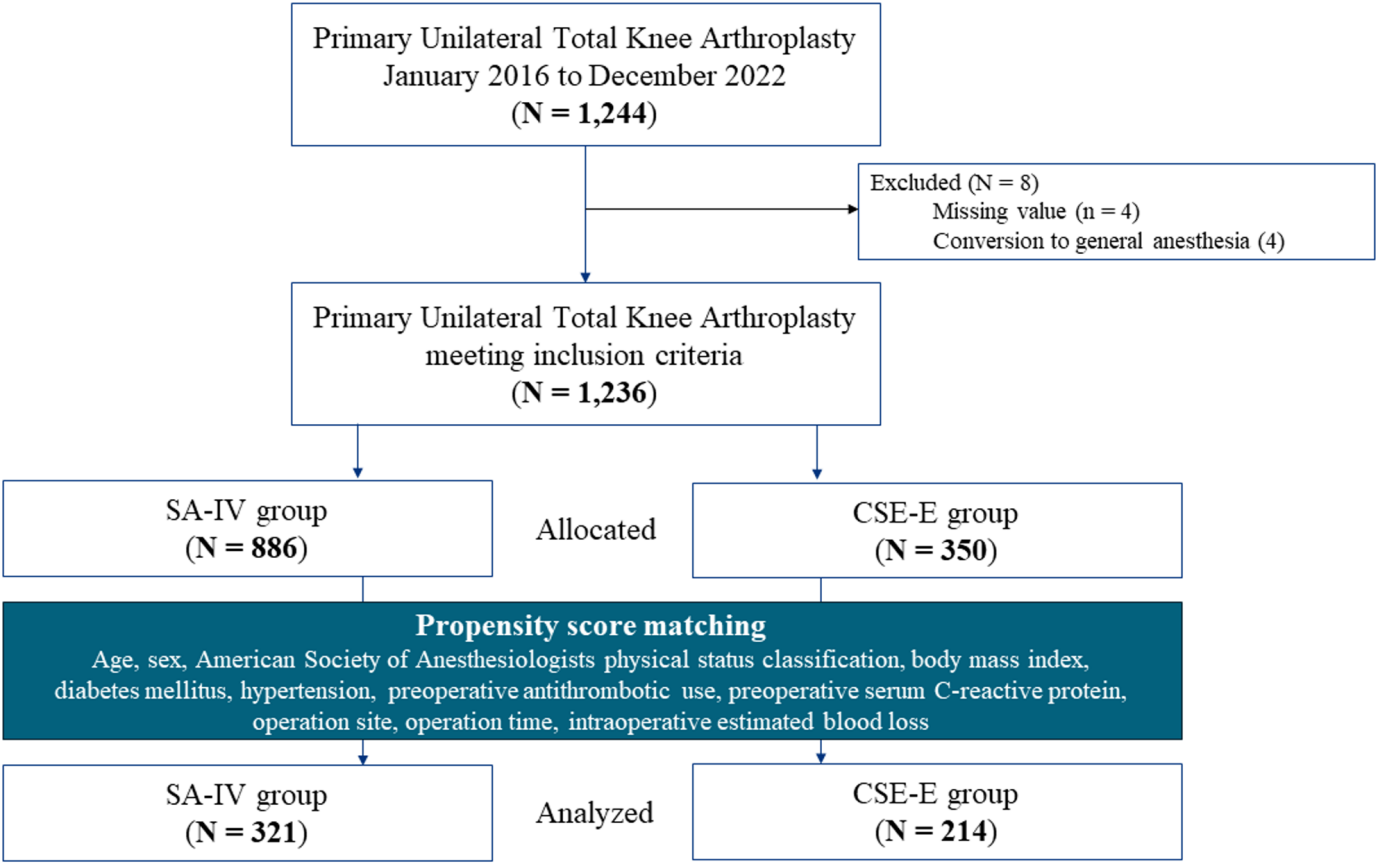
Study flow diagram. Abbreviations: SA-IV: Spinal anesthesia and intravenous patient-controlled analgesia, CSE-E: Combined spinal epidural anesthesia and epidural patient-controlled analgesia

**Fig. 2:**
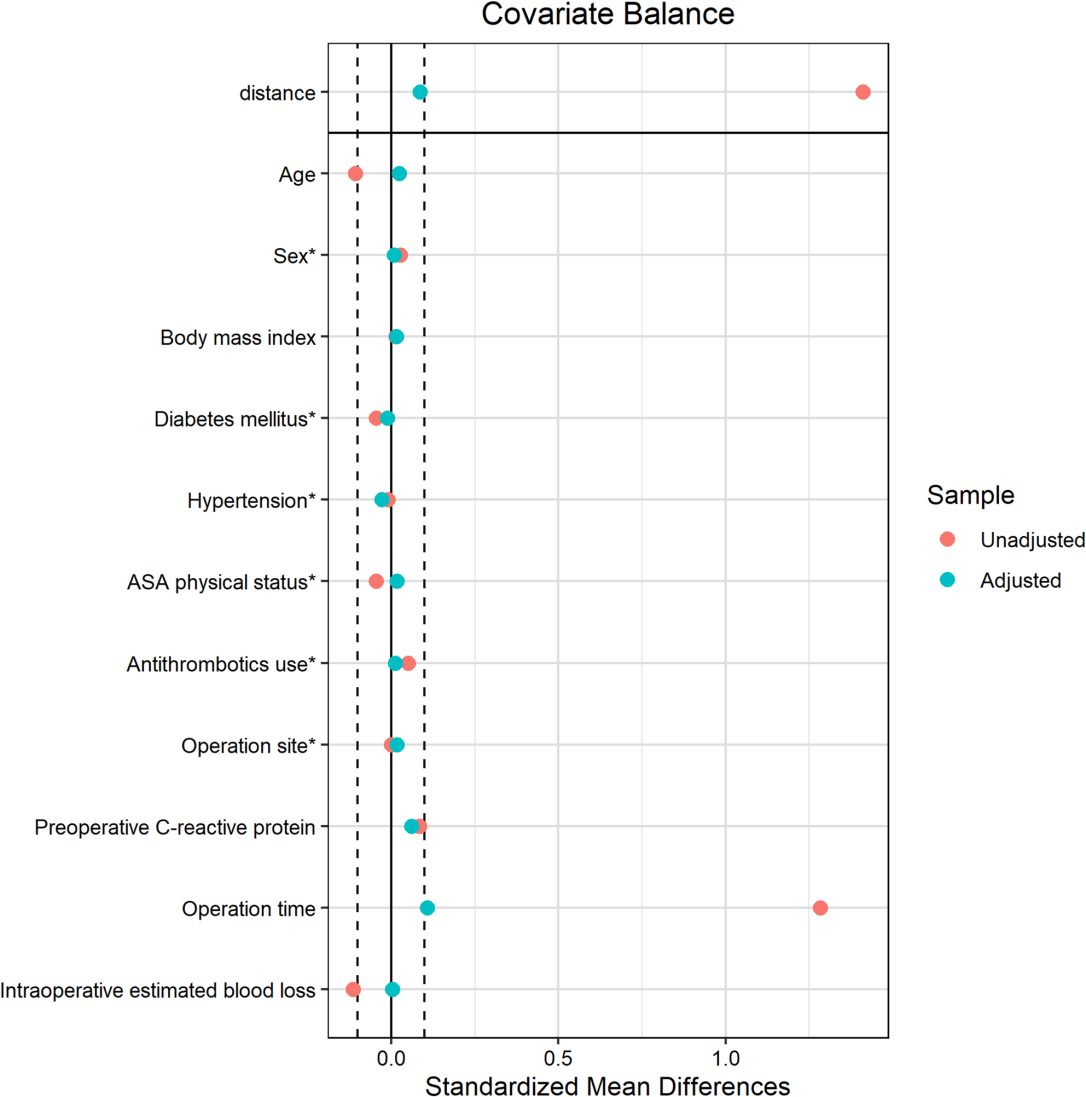
Love plot of propensity score matching based on the propensity score model. Standardized mean differences for matched data are smaller than 0.15. ASA = American Society of Anesthesiologists.

Of 1244 eligible patients, 1236 were included in analysis after excluding cases for missing data (*n* = 4) and conversion to general anesthesia (*n* = 4). Of the 1236 patients, 886 (71.7%) were in the SA-IV group and 350 (28.3%) were in the CSE-E group (Fig. 1). Table 1 shows the baseline perioperative characteristics of the unmatched and matched groups. Before matching, the distributions between the two groups were imbalanced in preoperative antithrombotic consumption, operation duration, and intraoperative estimated blood loss. After 2:1 nearest neighbor matching without replacement, patient characteristics were well balanced between the two groups, and 321 and 214 patients remained in the SA-IV group and the CSE-E group, respectively (Table 1; Fig. 2).

**Table 1:** Baseline characteristics of TKA patients before and after matching

Preoperative		Entire population				Matched population		
		SA-IV (*n* = 886)	CSE-E (*n* = 350)	*p*-value	SMD	SA-IV (*n* = 321)	CSE-E (*n* = 214)	SMD
Age	year	71.5 [67.0;76.0]	71.0 [66.0;76.0]	0.13	-0.1076	71.0 [66.0;76.0]	71.0 [66.0;75.0]	0.0235
Sex	female	717 (80.9%)	293 (83.7%)	0.289	0.0755	266 (82.9%)	178 (83.2%)	0.0253
BMI	kg/m^2^	26.4 [24.4;28.8]	26.6 [24.5;28.6]	0.772	0.0142	26.2 [24.0;28.6]	26.4 [24.4;28.4]	0.0155
DM		245 (27.7%)	81 (23.1%)	0.121	-0.1069	85 (26.5%)	53 (24.8%)	-0.0277
HTN		603 (68.1%)	235 (67.1%)	0.808	-0.0195	221 (68.8%)	141 (65.9%)	-0.0597
ASA physical status	I, II	732 (82.6%)	305 (87.1%)	0.062	-0.1352	283 (88.2%)	188 (87.9%)	0.0489
	≥III	154 (17.4%)	45 (12.9%)			38 (11.8%)	26 (12.1%)	
Antithrombotic consumption		110 (12.4%)	61 (17.4%)	0.027	0.1322	46 (14.3%)	34 (15.9%)	0.0308
C-reactive protein	mg/dl	0.1 [0.0;0.2]	0.1 [0.0;0.2]	0.196	0.0847	0.1 [0.0;0.2]	0.1 [0.0;0.2]	0.0605
Intraoperative								
Operation site	right	435 (49.1%)	172 (49.1%)	1	-0.0009	169 (52.6%)	110 (51.4%)	0.0327
	left	451 (50.9%)	178 (50.9%)			152 (47.4%)	104 (48.6%)	
Operation duration	minutes	75.0 [68.0;83.0]	101.0 [88.0; 114.0]	< 0.001	1.2835	86.0 [79.0;94.0]	91.0 [82.0;100.0]	0.1087
Estimated blood loss	milliliter	50.0 [30.0;50.0]	50.0 [30.0;50.0]	0.021	-0.1136	50.0 [30.0;50.0]	50.0 [30.0;50.0]	0.0032

Data are presented as median [interquartile range] or number (percentage)

*SA-IV* Spinal anesthesia and intravenous patient-controlled analgesia (PCA) group, *CSE-E* Combined spinal-epidural anesthesia and epidural PCA group, *SMD* Standardized mean difference, *BMI* Body mass index, DM, diabetes mellitus, *HTN* Hypertension, *ASA* American Society of Anesthesiology

### Primary outcome

The composite incidence of in-hospital TE events (myocardial infarction, ischemic stroke, pulmonary embolism, and deep vein thrombosis) was 0.7% (6/886) in the SA-IV group and 2.9% (10/350) in the CSE-E group (*p* = 0.004). In the matched population, the incidence was 0.9% (3/321) and 2.8% (6/214) in the SA-IV group and the CSE-E group, respectively, showing no significant difference (OR 1.88, 95% CI 0.89–10.57; *p* = 0.077). For double robustness, analysis with additional adjustment of propensity score was conducted, and a similar result was obtained [OR 1.92, 95% CI 0.91–11.74; *p* = 0.069] (Table 2).

**Table 2:** Incidence of in-hospital thromboembolic events

Variable	Entire population			Matched population							
	SA-IV (*n* = 886)	CSE-E (*n* = 350)	*p*-value	SA-IV (*n* = 321)	CSE-E (*n* = 214)	*p*-value	OR		Lower 95% CI		Upper 95% CI
Any thromboembolic event	6 (0.7%)	10 (2.9%)	0.004	3 (0.9%)	6 (2.8%)	0.077	1.882		0.885		10.565
Myocardial infarction	1 (0.1%)	2 (0.6%)	0.195	1 (0.3%)	2 (0.9%)						
Ischemic stroke	0 (0%)	2 (0.6%)	0.08	0 (0.0%)	1 (0.5%)						
Pulmonary embolism	2 (0.2%)	4 (1.1%)	0.057	1 (0.3%)	1 (0.5%)						
Deep vein thrombosis	4 (0.5%)	4 (1.1%)	0.233	1 (0.3%)	2 (0.9%)						
Any thromboembolic event^a^				3 (0.9%)	6 (2.8%)	0.0691	1.919		0.912		11.744

Data are presented as number (percentage)

*SA-IV* Spinal anesthesia and intravenous patient-controlled analgesia (PCA) group, *CSE-E* Combined spinal-epidural anesthesia and epidural PCA group, *OR* Odds ratio, *CI* Confidence interval

^a^Double robustness result by conducting an additional adjustment for propensity score

### Postoperative recovery

The median length of postoperative hospital stay was 7 days in both groups. The rates of postoperative transfusion and intensive care unit (ICU) admission were not different between groups. Peak NRSes on POD1 and ROM1 were higher in the SA-IV group [5 (3–6) vs. 3 (2–5), *p* < 0.0001; 5 (3–6) vs. 4 (2–5), *p* < 0.0001; respectively]. The median days to initiation of ROM exercise were 2 days and 3 days in the SA-IV group and the CSE-E group, respectively (*p* < 0.0001). In the subgroup of patients who had stopped antithrombotics perioperatively, the median day to resumption of medication was longer in the CSE-E group than in the SA-IV group [1 (1–1) vs. 4 (4–5); not statistically compared due to being subgroup] (Table 3).

**Table 3:** Postoperative recovery and complications

Variable	Entire population			Matched population					
	SA-IV (*n* = 886)	CSE-E (*n* = 350)	*p*-value	SA-IV (*n* = 321)	CSE-E (*n* = 214)	*p*-value	OR	Lower 95% CI	Upper 95% CI
Postoperative recovery									
Length of hospital stay after operation (days)	7 [7;7]	7 [7;9]	< 0.001	7 [7;7]	7 [7;9]	< 0.0001			
Postoperative transfusion	458 (51.7%)	218 (62.3%)	0.001	223 (69.5%)	146 (68.2%)	0.7462	1.197	0.664	1.342
Peak NRS on POD1	5 [3;6]	3 [2;5]	< 0.001	5 [3;6]	3 [2;5]	< 0.0001			
Peak NRS on ROM1	5 [4;6]	4 [2;5]	< 0.001	5 [3;6]	4 [2;5]	< 0.0001			
Days to the first ROM exercise	2 [2;2]	3 [3;4]	< 0.001	2 [2;3]	3 [2;4]	< 0.0001			
ICU admission	4 (0.5%)	2 (0.6%)	0.678	2 (0.6%)	2 (0.9%)	0.6852	2.740	0.209	10.854
Days to antithrombotic resumption^a^	1 [1;1]	4 [4;5]	< 0.001	1 [1;1]	4 [4;5]	-			
Postoperative complication									
Delirium	31 (3.5%)	21 (6.0%)	0.069	11 (3.4%)	12 (5.6%)	0.1923	1.485	0.772	3.632
Care-related (falls, bed sores)	5 (0.6%)	4 (1.1%)	0.282	2 (0.6%)	2 (0.9%)	0.6857	2.745	0.208	10.886
Metabolic (liver/kidney injury)	27 (3.0%)	10 (2.9%)	1	9 (2.8%)	7 (3.3%)	0.7436	1.626	0.452	3.039
Pulmonary	34 (3.8%)	6 (1.7%)	0.085	13 (4.0%)	4 (1.9%)	0.175	1.798	0.143	1.425
Cardiac	16 (1.8%)	9 (2.6%)	0.524	6 (1.9%)	3 (1.4%)	0.6831	2.047	0.183	3.040
New onset atrial fibrillation	4 (0.5%)	3 (0.9%)	0.411	2 (0.6%)	1 (0.5%)	0.8139	3.417	0.067	8.324
Unwanted neurologic symptom	18 (2.0%)	125 (35.7%)	< 0.001	8 (2.5%)	75 (35.0%)	< 0.0001	1.454	10.141	43.943
Lower extremity paresthesia	5 (0.6%)	15 (4.3%)	< 0.001	2 (0.6%)	9 (4.2%)	0.0138	2.205	1.487	32.980
Transient motor weakness	5 (0.6%)	62 (17.7%)	< 0.001	1 (0.3%)	36 (16.8%)	< 0.0001	2.695	9.271	451.782
Lower extremity numbness	10 (1.1%)	87 (24.9%)	< 0.001	4 (1.2%)	52 (24.3%)	< 0.0001	1.648	9.551	67.755
Back pain	8 (0.9%)	4 (1.1%)	0.749	5 (1.6%)	2 (0.9%)	0.5398	2.324	0.114	3.114
Others^b^	2 (0.2%)	4 (1.1%)	0.057	1 (0.3%)	2 (0.9%)	0.3686	3.417	0.272	33.559
Side effects of patient-controlled analgesia									
Nausea/vomiting	104 (11.7%)	52 (14.9%)	0.164	37 (11.5%)	35 (16.4%)	0.1102	1.289	0.912	2.470
Pruritus	114 (12.9%)	20 (5.7%)	< 0.001	40 (12.5%)	11 (5.1%)	0.0087	1.445	0.185	0.783
Urinary retention	52 (5.9%)	56 (16.0%)	< 0.001	17 (5.3%)	33 (15.4%)	0.0001	1.365	1.770	6.004
Sedation	28 (3.2%)	19 (5.4%)	0.087	13 (4.0%)	11 (5.1%)	0.5578	1.531	0.557	2.960
Hypotension	16 (1.8%)	21 (6.0%)	< 0.001	7 (2.2%)	16 (7.5%)	0.0065	1.606	1.433	9.169
Respiratory depression	15 (1.7%)	9 (2.6%)	0.436	8 (2.5%)	6 (2.8%)	0.8272	1.740	0.381	3.344
Dizziness	18 (2.0%)	22 (6.3%)	< 0.001	7 (2.2%)	11 (5.1%)	0.0609	1.606	0.960	6.153

Data are presented as median [interquartile range] or number (percentage)

*SA-IV* Spinal anesthesia and intravenous patient-controlled analgesia (PCA) group, *CSE-E* Combined spinal-epidural anesthesia and epidural PCA group, *OR* Odds ratio, *CI* Confidence interval, *NRS* Numerical rating scale, *POD1* first postoperative day, *ROM* first day of range of motion exercise, *ICU* Intensive care unit. Pulmonary complications included pneumonia, pulmonary edema, pleural effusion, and atelectasis. Cardiac complications included heart failure, cardiogenic shock, and new onset arrhythmia

^a^In the unmatched population, 110 and 47 patients in SA-IV and CSE-E group, respectively, had stopped preoperative antithrombotics and resumed postoperatively before discharge. After matching, total 74 patients remained, but not statistically compared due to being subgroup

^b^Other neurologic symptoms included headache and lower extremity pain

### General postoperative and neurologic complications

In the matched population, there were no between-group differences in the incidences of delirium; care-related problems (falls or bedsores); or metabolic (renal/hepatic insufficiency or failure), pulmonary (pneumonia, pulmonary edema, pleural effusion, atelectasis), and cardiac complications (cardiac arrest, heart failure, new onset arrhythmia, and new onset atrial fibrillation). The composite incidence of neurologic symptoms was significantly higher in the CSE-E group [*n* = 8/321 (2.5%) vs. *n* = 75/214 (35.0%); OR 1.45, 95% CI 10.14–43.94; *p* < 0.0001], particularly for paresthesia, motor weakness, and numbness (Table 3).

### Side effects of patient-controlled analgesia

The incidence of pruritus was higher in the SA-IV group [*n* = 40/321 (12.5%) vs. *n* = 11/214 (5.1%); *p* = 0.009). Conversely, urinary retention and hypotension occurred more frequently in the CSE-E group [*n* = 17/321 (5.3%) vs. *n* = 33/214 (15.4%); *p* < 0.001, *n* = 7/321 (2.2%) vs. *n* = 16/214 (7.5%); *p* = 0.007, respectively] (Table 3).

## Discussion

### Thromboembolic events

In this retrospective PSM analysis, no statistically significant difference in thromboembolic events was observed between intravenous and epidural analgesia after primary unilateral TKA under neuraxial anesthesia. We also found that patients receiving epidural analgesia had higher incidence of transient motor weakness, urinary retention, hypotension, more days to exercise initiation, but lower static and dynamic NRS pain scores than those receiving intravenous analgesia.

The novelty of this study lies in the comparison of venous and arterial thromboembolic events between analgesic techniques after neuraxial anesthesia for TKA. There is a scarcity of information on techniques used after neuraxial anesthesia compared with the abundance of studies comparing epidural to systemic analgesia after general anesthesia. While these studies ushered in the era of regional anesthesia-analgesia for orthopedic surgeries, they suffered limited relevance to current practice.

In several past studies, evidence regarding the impact of analgesic techniques on TE events generally favored epidural analgesia over systemic opioids (Moraca et al. 2003; Tuman et al. 1991; Modig et al. 1983). Epidural analgesia is proposed to mitigate the hypercoagulable state induced by postsurgical stress response through increased venous blood flow, reduced procoagulant and platelet activities, and anticoagulant effects of local anesthetics (Moraca et al. 2003; Hahnenkamp et al. 2002). Such effects have been demonstrated as reduced deep vein thrombosis, pulmonary embolism, and myocardial infarction in clinical studies (Rodgers et al. 2000; Kehlet and Holte 2001). However, these findings are based on comparison of epidural analgesia to systemic opioid-based analgesia after general anesthesia, which has largely been replaced by regional anesthesia in hip and knee arthroplasties. The ICAROS group recommends neuraxial anesthesia over general anesthesia in lower extremity arthroplasty based on a systematic review and meta-analysis (Memtsoudis et al. 2019), but differences in analgesic methods were not considered in their study. In a PSM cohort study comparing spinal to general anesthesia for total hip and knee arthroplasties with no differences in analgesic methods, spinal anesthesia showed lower incidence of 30-day mortality, but the incidence of pulmonary embolism was the same (Perlas et al. 2016).

Actually, a follow-up analysis of the ICAROS group revealed that peripheral nerve block (anesthesia/analgesia) was associated with lower risk for thromboembolism in TKA compared with no use. And the expert consensus emphasized the significance of peripheral nerve block as an analgesia adjunct with respect to TE complication rates (Stavros et al. 2021). With the widespread introduction of enhanced recovery after surgery protocols including multimodal analgesia (Aidan et al. 2021) and pharmacologic thromboprophylaxis, any protective benefit afforded by epidural analgesia may have been negated (Stavros et al. 2021; Luo et al. 2025; Dillane and Colleen Harnett 2025).

In our study, some perioperative features of the CSE-E group such as higher rate of lower extremity weakness, more days to ROM exercise initiation, and longer interruption of antithrombotics seemed to increase susceptibility to thromboembolism. Therefore, we initially suspected these characteristics to potentiate the negative effects of CSE-E.

Motor weakness is a common side effect of lumbar PCEA, with an incidence of 29.9 to 40%, resulting from anesthetization of lumbar and sacral motor fibers (Konigsrainer et al. 2009; Wu and Richman 2007; Hermanides et al. 2012; Ahmed and Baig 2016). However, such weakness is mostly transient and mitigated by lowering the infusion rate and/or adjusting the catheter tip location. Medical personnel monitor patients regularly for early detection of neurologic deficits and minimization of venous stasis. In the current study, we did not observe any cases of permanent sequelae among the patients with neurologic symptoms.

The median days to ROM exercise initiation was one day longer in the CSE-E group. However, the clinical significance of such a difference is uncertain because the definitions of early mobilization and rehabilitation after knee arthroplasty are heterogeneous, ranging from within 1 day to 1 week (Chen et al. 2012; Nakao et al. 2010; Lisi et al. 2017; Davila Castrodad et al. 2019).

The American Society of Regional Anesthesia guidelines on periprocedural antithrombotic use specify when to resume medications after epidural catheter removal (Horlocker et al. 2018). Despite such guidelines, it remains unclear as to how long patients at TE risk can safely maintain epidural catheters, and indwelling duration is at the discretion of attending physicians based on multiple factors including analgesic demand and TE risk. Furthermore, TE tendency varies by race. For example, the Asian race (all patients in this study) are known for a lower risk of thromboembolic events. The incidence of venous thromboembolism has been reported to be as low as 1% under mechanical prophylaxis alone in this race (Wong et al. 2022; Bin Abd Razak et al. 2012, 2017). The impact of interruption period on TE events in this study population remains unclear.

Therefore, our results may be explained by competition between the advantages and disadvantages of epidural analgesia compared with intravenous opioid-based analgesia.

### Postoperative pain relief and other complications

Analgesic efficacy of epidural analgesia has been reiterated in many studies (Choi et al. 2003; Meng et al. 2017). In line with these findings, the lower peak NRSes on POD1 and ROM1 in the CSE-E group in our study indicated PCEA to be more effective than IV-PCA for managing both static and dynamic pain. Pain control after TKA is paramount for postoperative rehabilitation, and its efficacy has the potential to manifest as better functional outcomes such as knee range of motion angles and patient satisfaction scores (Lavand’homme et al. 2022). Whether the greater pain relief provided by PCEA led to such benefits could not be determined in this study due to its retrospective nature.

In patients with neurologic changes, consultation with the anesthesiology department was necessary to assess and adjust the catheter tip location under fluoroscopy. Because catheter tip location can affect neurologic symptoms, the expertise of the anesthesiologist and assistance with the imaging modality are valuable, as represented by our lower incidence than those in previous studies (Ahmed and Baig 2016; Su et al. 2019). In our study population, two cases of back pain were due to epidural infection, one of which required surgical removal of an abscess. Neither led to permanent sequelae.

We observed a higher incidence of pruritus in the SA-IV group, which can be explained by the fentanyl-based IV-PCA regimen as opposed to a low-dose ropivacaine- and hydromorphone-based PCEA regimen (Ganesh and Maxwell 2007). Urinary retention and hypotension, two side effects more frequently observed in the CSE-E group, can occur from detrusor dysfunction and sympathetic blockade after epidural anesthesia and analgesia, respectively (Veering 2003; Choi et al. 2012).

### Limitations

There are several limitations in this study. First, our study population was primarily patients undergoing unilateral TKA conducted at a single center, limiting the generalizability of our results to other types of TKA like revisions and bilateral TKAs. These surgeries typically require longer operation time, immobile period, and epidural catheter indwelling duration, which can confound the incidence of TE events. Several observational studies have also reported that bilateral surgery and cemented prosthesis are risk factors for mortality after major joint replacement (Memtsoudis et al. 2009, 2011). Second, the incidence of thromboembolic events was very low. Although we did not find a statistically significant difference in TE complications between the two groups, the wide confidence intervals observed in our analysis indicate precision limitations due to the low event rate. Therefore, our results should be interpreted with caution, and we cannot rule out the possibility of a clinically relevant difference that this study was underpowered to detect. Third, confounding factors could not be eliminated due to the retrospective design, and bias may exist. For example, bias might have resulted from the influence of surgeon and anesthesiologist on the choice of anesthesia/analgesia. The surgeon could have requested use of CSE in surgically difficult cases because they expected long operations. Anesthesiologists might have chosen spinal anesthesia if difficult epidural catheter insertion was anticipated or had failed. Before PSM, significantly longer operation duration was observed in the CSE-E group, and more than half of the venous TE cases were excluded after matching. This suggests that prolonged operation may be associated with venous thromboembolism, as often reported, and that both surgical and anesthetic-analgesic factors should be considered (Hernandez et al. 2012). And, a subset of patients, especially in failed CSE cases in the operating room, had different start times for PCEA administration. This might introduce a confounding factor in the observations as it was a significant additional postoperative intervention.

Fourth, the etiology of neurologic symptoms was not collected in all cases, so those resulting from surgical complications such as peroneal nerve palsy could not be differentiated. However, neuropathy from surgical injury is uncommon (0.01% to 1.3%), and its incidence does not differ according to anesthesia type (Jacob et al. 2011; Yacub et al. 2009; Nercessian et al. 2005). Last, other less invasive options of regional analgesia such as femoral nerve block and adductor canal block have gained popularity and may be used in place of or as an adjunct to conventional methods. Despite this, epidural analgesia is still the gold standard for obstetric analgesia and is increasingly used in management of cancer pain. Our study provides useful insights on current and future practices (Heo et al. 2014).

## Conclusions

In this retrospective study, no statistically significant difference in thromboembolic events was observed between intravenous and epidural analgesia after primary unilateral TKA under neuraxial anesthesia. Despite the retrospective nature and its limited sample size, our result may provide a relevant link to the existing literatures regarding epidural vs. intravenous analgesia in TE complications after TKA. PCEA after CSE had better analgesic efficacy, but it was associated with higher incidences of side effects including motor weakness, urinary retention, and hypotension. Most TKA patients have multiple risk factors for thromboembolism such as old age, female sex, obesity, and postoperative immobility. Therefore, the choice of analgesia for these patients should be based on risk-and-benefit assessment tailored to medical and surgical conditions reflecting recent recommendations.

## Data Availability

The data for the present study is available from the corresponding author on reasonable request.

## Abbreviations

TKA: Total knee arthroplasty
TE: Thromboembolic
SA: Spinal anesthesia
CSE: Combined spinal and epidural anesthesia
IV-PCA: Intravenous patient-controlled analgesia
PCEA: Epidural patient-controlled analgesia
SA-IV: Spinal anesthesia and intravenous patient-controlled analgesia
CSE-E: Combined spinal and epidural anesthesia and epidural patient-controlled analgesia
POD: Postoperative day
ROM: Range of motion
